# Imagery in the entropic associative memory

**DOI:** 10.1038/s41598-023-36761-6

**Published:** 2023-06-12

**Authors:** Luis A. Pineda, Rafael Morales

**Affiliations:** 1 Universidad Nacional Autónoma de México, IIMAS, Mexico, 04510 Mexico; 2 Universidad de Guadalajara, SUV, Guadalajara, 44130 Mexico

**Keywords:** Psychology, Mathematics and computing

## Abstract

The Entropic Associative Memory is a novel declarative and distributed computational model of associative memory. The model is general, conceptually simple, and offers an alternative to models developed within the artificial neural networks paradigm. The memory uses a standard table as its medium, where the information is stored in an indeterminate form, and the entropy plays a functional and operation role. The memory register operation abstracts the input cue with the current memory content and is productive; memory recognition is performed through a logical test; and memory retrieval is constructive. The three operations can be performed in parallel using very few computing resources. In our previous work we explored the auto-associative properties of the memory and performed experiments to store, recognize and retrieve manuscript digits and letters with complete and incomplete cues, and also to recognize and learn phones, with satisfactory results. In such experiments a designated memory register was used to store all the objects of the same class, whereas in the present study we remove such restriction and use a single memory register to store all the objects in the domain. In this novel setting we explore the production of emerging objects and relations, such that cues are used not only to retrieve remembered objects, but also related and imaged objects, and to produce association chains. The present model supports the view that memory and classification are independent functions both conceptually and architecturally. The memory system can store images of the different modalities of perception and action, possibly multimodal, and offers a novel perspective on the imagery debate and computational models of declarative memory.

## Introduction

The Entropic Associative Memory (EAM)^1–3^ is a novel computational memory model in which functions representing arbitrary concrete or abstract objects are stored in a bi-dimensional array or table, called Associative Memory Register (AMR), which is used as the representational medium. The columns and the rows stand for the arguments and their values, respectively, and the functional relation is represented by filling up the cell at the corresponding intersection, for all the columns. Hence, every object is stored by marking up one cell of each column in the AMR, and can be thought of as a memory trace. In this section we illustrate intuitively the structure and functionality of the model; its evolution from its original presentation; and state the goals and experiments of the present investigation.

### Intuitive illustration of the EAM model

**Figure 1:**
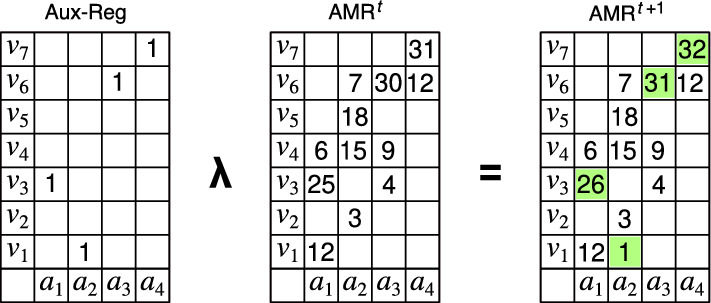
Illustration of the $\lambda$-register operation. The function $\{(a_1, v_3),(a_2, v_1),(a_3, v_6),(a_4,v_7)\}$ representing the cue is placed in table format in the auxiliart register (Aux-Reg) and then registered in a the Associative Memory Register (AMR) which already has some content at the state *t*. The value of each cell used by the cue is added to its corresponding cell in the AMR at state *t* yielding the state $t+1$.

**Figure 2:**
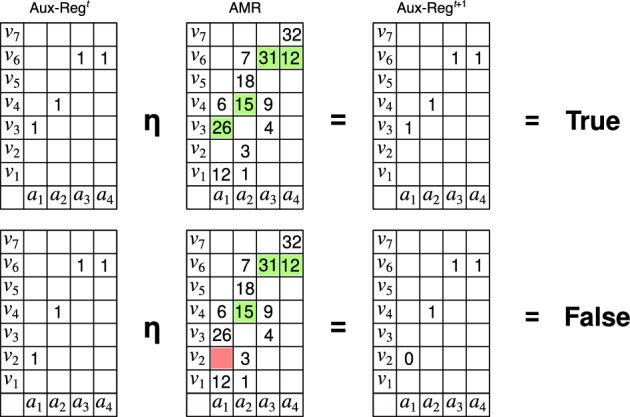
Illustration of the $\eta$-recognition operation. In the top diagram the cue is accepted as all the cells of the AMR corresponding to the cells used by the cue in the auxiliary register have weights different from zero. The result of the operation is shown in the auxiliary register at state $t+1$ at the right, in which all cells have a value of 1—for clarity only the cells used by the cue are shown. The bottom diagram shows the opposite case in which the cue is rejected, as there are cells in the AMR corresponding to cells used by the cue whose values are zero. The result of the operation is shown in the auxiliary register at state $t+1$ in which the value of one cell is zero.

**Figure 3:**
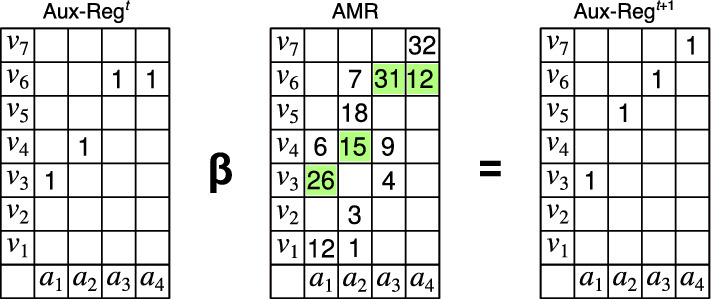
Illustration of the $\beta$-retrieval Operations. Each column of the auxiliary register is considered a normal probability distribution centered at the actual cell used by the cue, with standard deviation $\sigma$, which is a parameter of the operation. The columns of the AMR are also considered probability distributions, and the value retrieved for each column of the AMR is selected randomly from the product of the distribution representing the cue and the distribution of the AMR’s corresponding column.

The EAM model defines three memory operations in relation to a cue, which are called $\lambda$-register, $\eta$-recognition and $\beta$-retrieval. The functions representing the inputs and the outputs are placed on another table, called the auxiliary register, with the same dimensions of the AMR. The $\lambda$-register is defined as the logical disjunction^1,2^ or the addition^3^ between the value of each cell in the auxiliary register and the value of the corresponding cell in the AMR; the $\eta$-recognition is defined through the logical material implication between the cells in the auxiliary register and the corresponding cells in the AMR, so the operation is true if the cue is included in the memory and false otherwise; and the $\beta$-retrieval selects a row of the AMR that corresponds to the value of the retrieved object, for all the cells used by the cue. These operations are illustrated diagrammatically in Figs. 1, 2 and 3. The $\lambda$-register and $\eta$-recognition operations are cell-to-cell operations and can be performed in parallel if the appropriate hardware is provided. Likewise, $\beta$-retrieval is a column-to-column operation that can also be performed in parallel.

**Figure 4:**
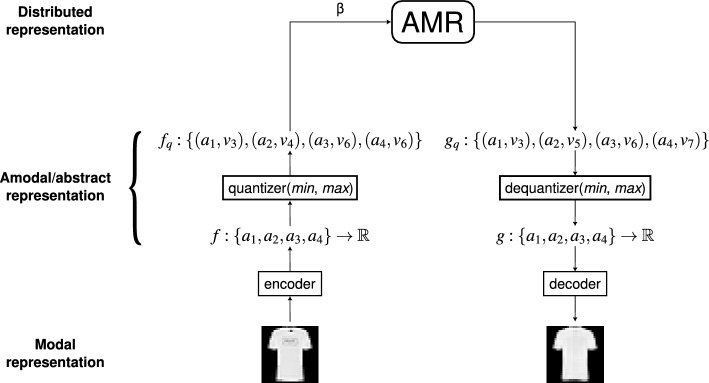
Illustration of the levels of representation. The lower representational level is constituted by modality-specific buffers that hold the input and output cues, in the present case pieces of clothing of the Fashion-NMIST Corpus. The input concrete images are mapped by the coder into functions whose arguments correspond to the columns of the AMRs. These functions constitute abstract amodal representations of the input images at the second or intermediate level of representation. The functions are mapped back into their corresponding concrete images by the decoder. The quantized functions are the objects that are the subject of the memory operations and are included in the distributed representation at the top level.

The functions that are input to and output from the memory constitute abstract amodal representations of the stored modality-specific concrete images. These are placed in input and output buffers, such as pixel buffers in the case of visual images^1,2^ and MFCC vectors in the case of phonetic information^3^. Such concrete images are mapped into their corresponding functions and vice versa through a coder and a decoder, respectively. The output of the coder and the input to the decoder are real values, but they are sampled into discrete levels that correspond to the number of rows of the AMR. The whole of the memory system includes three representational levels: the first or bottom level in which the input and output concrete images are represented; the second or intermediate level where their corresponding abstract amodal representations as finite discrete functions with discrete values are placed; and the third or top level where the distributed representation is stored in the AMR. The three levels of representation are illustrated in Fig. 4. The image is a piece of clothing taken from the Fashion-MNIST data set^4^, as explained below.

### Evolution of the EAM model

The basic EAM^1,2^ uses AMRs with boolean values. The $\lambda$-register and the $\eta$-recognition operation are implemented directly with the logical inclusive disjunction and the material implication between cells of the auxiliary register holding the cue and the corresponding cells of the AMR, respectively. In addition to the overtly input cues, the distributed representation allows for the emergence of new functions or memory traces conformed by combinations of cells whose value is 1 by taking one cell of each column at a time. The emerging objects can be thought of as representations of potential objects related to the cues, and can be used for recognizing novel inputs and for constructing novel outputs. However, it is not possible to distinguish the overtly registered functions from the emerging ones, and the representation is *indeterminate*. Indeterminacy is measured with the entropy, and the memory has an entropy value at each state. Let $\nu _i$ be the number of non-zero cells in column *i*; if there are not cells with value 1 then $\nu _i=1$. The entropy $e_i$ of the column *i* is $log_2{\nu _i}$. The entropy *e* of the AMR is the average entropy of all columns. The number of functions stored at any given state in the AMR is the number of combinations that can be formed by selecting a cell whose value is 1 for each column at a time. Hence, the number of functions included in an AMR is $\prod _{i=1}^{n}\nu _{i}$ where *n* is the number of columns. However, this also the average number of marked cells of all columns to the power of the number of columns. Hence, $\prod _{i=1}^{n}\nu _{i} = (2^e)^n = 2^{en}$. This number is very large even for a small value of the entropy and a moderate number of columns, and allows for the construction of memories with a very large capacity and yet requiring a very limited amount of computing resources.

The $\beta$-retrieval operation constructs a novel object if the cue is accepted. For this, each column of the AMR is considered a probability distribution of the cells with non-zero values, and the cue is modeled as a triangular distribution centered in the cue’s actual value, for each column. The value retrieved for each argument is produced randomly from the product of the distribution representing the cue and the distribution of the column. Cues are determinate, even if they are incomplete, and $\beta$-retrieval produces determinate objects out of the cue and the indeterminate “memory mass”. The level of indeterminacy gives rise to *the entropy trade-off*: if the entropy is low the precision of the memory recognition and retrieval operations is high but the recall is low; conversely, if the entropy is high or very high, the recall is higher but the precision lowers; however, there is a range of moderate entropy values in which the precision and the recall are both high, and the memory has a satisfactory performance.

The basic EAM was used to store, recognize and retrieve reconstructions of objects using complete and severely occluded cues—e.g., manuscript digits^1^ and manuscript capital and lower-case letters^2^. A designated AMR was used for storing objects of the same class and cues were presented and recovered from all the AMRs, but memory register or learning was supervised. In such a setting cues are often accepted by more than one AMR and a system’s level mechanism to decide the right class of the stored object was defined, in addition to the basic memory operations. Such mechanism was simply to select the object produced by the AMR whose entropy was the lowest. Such architecture also required that the whole memory were constituted by a number of AMR using mutually exclusive local memory regions. However, we also showed that objects of different classes could be stored in the same memory register without degrading the performance of the system but at the expense of a small increment of the entropy. For instance, instead of using 10 memory registers to store the 10 digits we used only 5 registers, each including two digits, so the memory could retrieve a digit of one kind cued by a digit of a different kind^1^; and instead of storing all capital and lower case letters using 47 memory registers, we collapsed capital and lower case letters with different shapes into the same register, reducing the number of memory registers to 36, and the system could retrieve a capital letter cued by a lower-case one and vice versa^2^.

The next step in our research program was to introduce weights to the cells according to their frequency of use. This addition gave rise to the weighted model W-EAM^3^. In the new version the $\lambda$-register operation was defined as the arithmetic sum of the value of the cell used by the cue in the auxiliary register, which is 1, and the current value of its corresponding cell in the AMR, for all cells used by the cue. The operation reinforces all the cells used by the registered cue simultaneously and can be considered a form of Hebb’s learning rule. The entropy of each column becomes Shannon’s entropy directly, and the entropy of the memory as a whole is the average entropy of all the columns^3^. The number of functions or units of content held in the memory at any state, both overt and emerging, is still $2^{en}$ but corresponds to the functions that are likely to be recovered according to the indeterminate state of the memory rather than the total number of functions that can be constructed using all cells with non-zero values.

The weights of the W-EAM version allow to model the sensitivity of the memory and the strength of a cue. For this we introduced three parameters in the definition of the $\eta$-recognition operation called $\iota$, $\kappa$ and $\xi$. The parameter $\iota$ modulates the minimum weight that a cell of the AMR must have to be considered on; $\kappa$ controls the minimum average weight of the cells of the AMR hit by the cue, designed $\rho$, needed for the cue to be accepted. Large values of these parameters make harder that marginal cues are accepted, preventing false positives. However, this condition may be too strict and produce false negatives. This constraint is relaxed with the incorporation of the parameter $\xi$ stating that a number of columns may fail to accept a cue, and yet the cue as a whole is accepted. In this setting a cue may be accepted by more than one AMR and the system level selection mechanism chooses the object accepted by the AMR with the lowest value of the product of the entropy and $1/\rho$. The impact of these parameters is explored in the W-EAM model^3^; however, they are not the focus of the present study and in the experiments we use their default values, which impose no constraint to the $\eta$-recognition operation. In the W-EAM model the cue is modeled with a normal probability distribution with standard deviation $\sigma m$—where $\sigma$ is a parameter of the operation and *m* is the number of rows—centered at its actual value, for each column, such that the object constructed by the $\beta$-retrieval operation is selected randomly from the product of the distribution representing the cue and the distribution of the corresponding column. Hence, the parameter $\sigma$ models the degree of indeterminacy that is allowed in the construction. If $\sigma = 0$ the cue selects its own value and the memory becomes reproductive or photographic. The W-EAM was applied to store and learn Mexican Spanish phonetic information with satisfactory results, showing that the memory can store speech images in addition to the visual images studied in the initial experiments.

### Remembering variability and association chains

In this paper we take to the limit the capability of AMRs of overlapping arbitrary objects and use only one AMR to store all the objects of all classes. The resulting memory architecture overcomes the problem of partitioning the memory in local regions, optimizes the use of the medium, and learning is now unsupervised. The model demarcates explicitly the memory proper—all objects are stored in the medium independently of their classes—from the classifier, which operates on the input and output cues, and the memory and the classifier are independent modules, conceptually and architecturally. Objects are recognized and recovered in relation to complete or partial cues, and cues of one class can retrieve related objects of a different class, establishing genuine association chains. We consider that the act of remembering is performed in relation to a context, and that agents have an intent when retrieving information from memory, and may focus on remembering objects, but also on retrieving objects related to the cue, or even on imagining novel objects. Here, we model such functionality by modulating the parameter $\sigma$ of the $\beta$-retrieval operation, that controls the similarity of the rendered object in relation to the input cue. Lower values of $\sigma$ result on remembering, and the lower the value, the more reproductive the recollection; moderate values are oriented to recover objects related to the cue, perhaps of a different class, allowing the production of association chains; and large values allow the retrieval of objects that are rather different from the cue, may be assigned a different class, possible faint and vague, and yet can have an interpretation, which are referred to here as imaged objects.

The present extension of the theory has been tested using the Fashion-MNIST data set^4^ through five experiments, as follows:

1. *Experiment 1* Determine the optimal size of the memory register for the domain.
2. *Experiment 2* Determine the performance of the memory retrieval operation; illustrate objects recovered from the memory using complete cues of all 10 classes of Fashion-MNIST, for different values of the parameter $\sigma$.
3. *Experiment 3* Repeat (2) but using incomplete cues in the memory retrieval operations.
4. *Experiment 4* Produce association chains on the basis of complete cues; in this scenario the object recovered from the initial memory retrieval operation is used as the cue to a new retrieval operation, and the process is repeated recurrently.
5. *Experiment 5* Repeat (4) using incomplete cues to initiate the association chains.

The rest of this paper is structured as follows: in the second section we discuss the properties of associative memories and place the model in relation to the literature of the field; in the third we present the formal specification of the memory operations and in the fourth the system’s architecture, as well as the corpus used in the experiments; then we present the experiments and results and conclude with the general discussion.

## Properties of associative memories and related work

Computational models of associative memory have a number of properties that allow their characterization—although there is no universal agreement on their definition and scope. In the present framework these are understood as follows:

1. *Associative* The memory operations are performed relative to a cue. Associative memories are accessed by content and oppose memory systems that use memory registers with addresses, as the standard Random Access Memories (RAM) of digital computers.
2. *Distributed* The units of the memory medium can be used to store different concepts or units of content at the same time, and each unit of content can share units of the medium with other units of content. This is, the relation between the units of the medium and the units of content is *many-to-many*^5^. Hence, in distributed memories, different units of content can “overlapp” on the medium. This property opposes local memories in which memory locations or regions of the medium are mutually exclusive partitions, each assigned to store a specific content, such as standard RAM memories, or systems in which every unit of content is located at a particular region in some abstract space that does not intersect with other regions in such space.
3. *Declarative* The information is registered, recognized and retrieved through direct manipulations on the medium using very simple algorithms, which always terminate, and involve no search. Writing and reading information in RAM registers are declarative operations. This property opposes procedural memories which are trained, usually involving complex algorithms and very large numerical matrices. While objects in declarative memories are remembered, information in procedural memories is deployed. This opposition differs from the traditional distinction between symbolic representations—which are normally assumed as local—versus sub-symbolic—which are assumed as distributed—and allows for symbolic distributed representations.
4. *Abstractive* The memory register operations makes an abstraction of the cue and the whole of the memory content, and produces a new state of the memory holding such abstraction.
5. *Productive* There are interactions between the units of content explicitly input through the memory register operation, from which novel units emerge. These units allow the recognition of variants of the registered inputs and even of novel inputs, and the construction of novel outputs, but also may cause the production of false memories.
6. *Determinate* Specific units of information have a clear demarcation within the medium and can be identified by inspection directly; this property opposes *indeterminate* memories in which the units are intertwined within the memory medium or the representation space and their identity within the medium is dissolved.
7. *Entropic* Indeterminate memories have an amount of entropy; the larger the entropy the larger the indeterminacy; conversely, the entropy of a fully determinate memory is zero.
8. *Direct rejection* Cues not included in the memory are rejected through a direct test without search. This is a natural property of human memory, which poses a strong challenge to memory theories^6–9^. Systems that never reject a cue may retrieve its most similar object within the memory, but such object is a false positive in a strict sense. If rejection does occur, a form of the so-called Closed-World Assumption (CWA) of knowledge-based system, to the effect that if a proposition cannot be proven is considered false, is adopted implicitly^10^. However, the CWA only holds if the stored knowledge is complete, in the sense that there are not facts in the world that falsify the system’s response. In the memory setting, the assumption is that if the search process fails, the sought object is not included in the memory; however, this only holds if the cue is searched exhaustive, which is implausible if the memory is very large. In addition, whether the search process terminates cannot be predicted in the most general case due to the halting problem^11^.
9. *Constructive* The objects produced by the memory retrieval operation are dynamic constructions; retrieved objects may be modifications of the originally stored objects, but also objects associated to the cue, and even objects imaged on the basic of the cue and the stored objects. This property opposes reproductive of photographic memories that retrieve the cue object exactly as it was originally registered. RAM memories, for instance, are reproductive or photographic. Systems that lack the direct rejection property but are unable to reject a cue, retrieve the object most similar to the cue, and hence an association, but not a genuine construction.
10. *Capacity* The number of units of content that can be stored in the memory in relation to its structural and functional parameters. This parameter can be further specified into the number of objects that are stored in the memory at a particular state, and into the amount of stored information in which the system performs satisfactorily.

From the examples in Figs. 1, 2 and 3 and the previous discussion it can be readily seen that the EAM system is associative, distributed, declarative, abstractive, productive, indeterminate, entropic, supports direct rejection, is constructive and has a very large capacity, resembling the corresponding putative properties of human memory^12–15^. The EAM model is mostly consistent with the properties suggested for human associative memory in Hintzman’s framework and the Minerva system^16^, but such approach differs from the EAM in the representational format and the memory operations. In particular, although Minerva also uses a table as its representation medium, each new input is expressed as a new row, so the table grows with the number of stored objects. This opposes EAM in which the table is fixed, and the new inputs overlap with the current memory content.

### Relation of EAM to ANN models

Computational models of associative memory have been extensively studied within the Artificial Neural Networks (ANNs) paradigm. The literature on the subject is pretty abundant, and there are two main conceptualizations: as a dynamical system implemented with recurrent neural network, such as Hopfield’s model^17^ and related work^18–24^, and as a feedforward network^25–27^. Recurrent neural networks have been used for modeling working memory^28–30^. Autoencoders^31^ can also be considered associative memories, as these systems reproduce the input cue, although are trained with standard back-propagation, and updating the memory involves full retraining. It is argued that the class of memory models that use high dimensional vectors can be unified in a single ANNs framework^32–38^, even those that are not formulated as neural networks, such as Minerva^39^.

The main difference between the Entropic Associative Memory and ANNs models is that EAM is not an artificial neural network. Although both approaches represent objects through feature-value structures, in EAM these are interpreted as functions representing objects, as in symbolic representational systems; while in the ANNs framework such structures are interpreted as vectors in a high dimensions geometric space. The memory system do uses a coder, a decoder and a classifier, implemented with neural networks, but these modules constitute only the scaffolding supporting the memory structure and the input and output operations. For this EAM differs from of ANNs models in the way its properties are understood, and on the values of some of such properties. A punctual comparison between EAM and ANNs models in relation to the memory properties is beyond the scope of the present paper. However, at an intuitive level, EAM’s declarative specification of the three memory operations allows that the information is remembered and retrieved constructively, and opposes the procedural nature of most ANNs models, where the memory is trained. The memory system as a whole differs from architectures including a bottom sub-symbolic layer implemented with ANNs and a top symbolic layer implemented with logical or linguistics structures^40^ or RAM registers^41^, as the representation held in EAM is symbolic but also distributed. Also, the abstractive form of $\lambda$-registers supports a form of learning in which inputs are integrated directly into the memory mass of the distributed representation, where units of content are overlapped. Hence, learning corresponds to knowledge acquisition and may be unsupervised. In addition, the overlapping of units of content on the memory medium opposes most ANNs models which are rather focused on separating the input units in mutually exclusive points or regions of some abstract representation space. In Hopfield’s model, for instance, every object is stored in a local minimum of the energy function, so the representation of individual objects is fully determinate. The productivity of the memory is analogous to the generalization property of neural networks^5^ that give rise to classes, but not to individual novel instances, as in the present model. EAM’s notion of productivity is useful to recognizing novel inputs and enables the constructive character of the memory retrieval operation. It also may be useful for modeling the false recall phenomenon^42^ and may play a role in imagination^15^. The indeterminacy in EAM is due to the superposition of the represented units, and the entropy is a functional and operational parameter of the memory, in opposition to most ANNs models in which the entropy has no functional role or is not event used. The ability to reject directly cues not contained in the memory through a declarative test is neither normally found in ANNs memories, and the $\eta$-recognition operation may have empirical support^6–9^. Another distinctive feature of EAM is the bayesian nature of the $\beta$-retrieval operation, where both the cue and the memory are considered probability distributions, corresponding to a likelihood and a prior, respectively—where the degree of indeterminacy of the constructive process is controlled by a single parameter—has neither a clear counterpart in ANNs models. The constructive character of EAM opposes most ANNs models, as was mentioned above for Hopfield’s model, in which in memory retrieval recovers the stored object exactly. Such model is often argued to be constructive, but this perception is due to the use of incomplete cues which select complete objects, and their retrieval is seen as a construction. The reproductive character of Hopfield’s memory can be appreciated directly if only complete cues are used. Finally, the memory capacity of EAM is very large, its content at a given state is computed directly in terms of the entropy and the number of columns of the AMRs, and its operational range depends on the entropy trade-off. In addition, EAM uses a very small number of units of the memory medium; the memory operations are implemented with minimal algorithms that always terminate, and the relation of its capacity to its cost is very satisfactory.

## Memory operations

For the experiments we use the formal definition of the operations of the W-EAM system^3^ with the exception of the functionality for selecting the class of a cue at the system level, whenever there is more than one memory register accepting the cue, that is not required in the experiments below. For clarity and for making this paper self-contained, we include the explicit definition of the memory operations but specified to the case in which only one memory register is used.

Let the sets $A = \{a_1,\ldots ,a_n\}$ and $V = \{v_1,\ldots ,v_m\}$ be the domain and the codomain of a weighted relation $r: A\rightarrow V$ stored in the memory, and let the function $R: A \times V \rightarrow \{0,\ldots , l\}$, where *l* is an integer greater than zero, specify the weights of *r*, such that $R(a_i, v_j) = w_{ij}$ and $w_{ij} \ne 0$ if and only if $(a_i, v_j)$ is in the relation *r*. Let $r_f$ and $r_a$ be two arbitrary relations from *A* to *V*—which are held in the memory and in the auxiliary register, respectively; let $f_a$ a function with the same domain and codomain representing the cue; and $\Psi _i$ for $1 \le i \le n$ the probability distribution defined by the weights assigned to $(a_i,v_j)$, for all $1 \le j \le m$. The operations are defined as follows:

1. *Memory Register* $\lambda (r_f,r_a) = q$ such that $q = r_f \cup r_a$, so $Q(a_i,v_j) = R_f(a_i,v_j) + R_a(a_i,v_j)$ for all $a_i \in A$.
2. *Memory Recognition* $\eta (r_a,r_f,\iota ,\kappa ,\xi )$ is true if $R_a(a_i,v_j) \rightarrow g(R_f(a_i,v_j))$ for all $v_j$ for at least $n-\xi$ arguments $a_i$ of $r_f$ (i.e., material implication relaxed by $\xi$) and $\rho \ge \kappa \Omega$, and false otherwise, such that:
  - $\omega _i=\frac{1}{k}\sum _{j = 1}^mR_f(a_i,v_j)$ where *k* is the number of cells in column *i* in $r_f$ such that $w_{ij} \ne 0$; i.e., the average weight of the argument $a_i$ among all the none-zero cells in the column;
  - $g(R_f(a_i,v_j))=1$ if $R_f(a_i,v_j) \ge \iota \omega _i$, and 0 otherwise.
  - $\Omega = \frac{1}{n}\sum _{i = 1}^n \omega _i$; the average weight $\omega _i$ of all columns *i*.
  - $\rho =\frac{1}{n}\sum _{i = 1}^nR_f(a_i,v_{cue})$ where $v_{cue}$ is the value $= v_j$ of the argument $a_i$ in the cue $R_a(a_i,v_j)$; i.e., the weight of the cue.
3. Memory Retrieval $\beta (f_a,r_f,\sigma ) = f_v$ such that if $\eta (r_a,r_f,\iota ,\kappa ,\xi )$ holds $f_v(a_i)$ is some $v_j$ that is selected randomly from $r_f(a_i)$—i.e., the column *i*. Let $\zeta$ a normal distribution centered at the cue $f_a(a_i)$ with standard deviation $\sigma m$. Let $\Phi _i$ be the product of $\Psi _i$ and $\zeta _i$. $f_v(a_i)$ is selected randomly from $\Phi _i$ for all the arguments *i*. If $\eta (r_a,r_f,\iota ,\kappa ,\xi )$ does not hold then $\beta (f_a, r_f,\sigma )$ is undefined—i.e., $f_v(a_i)$ is undefined—for all $a_i$.

## System’s architecture

The memory system includes three representational levels from bottom to top—see Fig. 4—as follows:

1. The modal specific input and output representations of the domain. In the present study we use the corpus Fashion-MNIST^4^ of pieces of clothes, bags and shoes of ten classes, including 70,000 images of 28 $\times$ 28 pixels with 256 gray levels, as specified in Table 1. The corpus is balanced and there are 7000 images of each class.
2. The abstract amodal representations of the modal objects in the first level. These are finite discrete functions with *n* arguments with real values representing the corresponding concrete images. For this study, domains with cardinalities of 32, 64, 128, 256 and 512 were considered.
3. The abstract amodal distributed representation held in the memory. The memory has *n* columns as in (2) and *m* rows. The value of each argument at this level is the quantized value, in *m* levels, of the corresponding argument of the local representation in (2).

**Table 1:** Classes of the fashion-MNIST corpus^4^.

Class Id	Class name
0	T-shirt/top
1	Trouser
2	Pullover
3	Dress
4	Coat
5	Sandal
6	Shirt
7	Sneaker
8	Bag
9	Ankle boot

**Figure 5:**
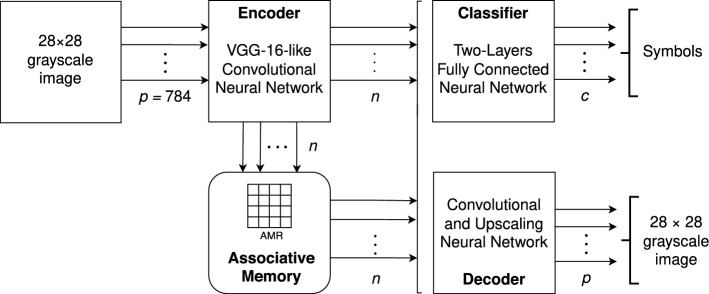
System architecture. The system has four modules: encoder, decoder, associative memory, and classifier. The encoder is a convolutional neural network based on VGG16^43^ with an input of $28 \times 28\times 1$ parameters ($p = 784$ in total), and an output of *n* values, where *n* is the number of columns of the unique associative memory register. The decoder is a convolutional and upscaling neural network that receives *n* parameters as input and generates a $28 \times 28$ grayscale image, whereas the classifier is a dense neural network with two layers that receives the same number of parameters as input and produces a probability distribution of $c = 10$ elements. The system as a single input, an image to the encoder, and two possible outputs, a classification and an image. The encoder can connect directly to the decoder and the classifier (e.g. for training and testing), but it is used mainly to feed the assocative memory, that in turn feeds the other two modules.

The system’s architecture is analogous to our previous work and includes a coder and a decoder mapping the modal and concrete representations at the first level to the abstract amodal representations at the second and vice versa; these modules are implemented with standard convolutional deep-neural networks—an encoder and a decoder, constituting and autoencoder. The architecture includes also a classifier, trained in conjunction with the encoder and the decoder, and used for classifying the outputs from the memory. The functional architecture is illustrated in Fig. 5.

We divided the original autoencoder into the coder and the decoder which were used as independent networks. In the original presentation of EAM^1,2^ the coder, the decoder and the classifier were trained together, and then we simply removed the classifier for the memory experiments proper. Next, in the W-EAM model^3^, we first trained the coder and the classifier; then the decoder, and for the memory experiments we used the three components independently. In particular, the classifier was used for enriching the phonetic corpus in incremental stages. Finally, in the current experiments we trained the three components together again, but used them as independent neural networks as their inputs and outputs can be communicated flexibly.

## Experiments and results

### Experiment 1

For the experiments the fashion-MNIST corpus was partitioned in three mutually exclusive partitions, as follows:

- Training Corpus *TrainCorpus*: For training the coder and the decoder, and the classifier (70%);
- Remembered Corpus *RemCorpus*: For filling up the memory (20%);
- Test Corpus *TestCorpus*: For testing the full classifier (encoder-classifier), the autoencoder (encoder-decoder), and the memory (10%).

The memory system was set up using the following procedure:

1. Train the classifier and the autoencoder simultaneously with the *TrainCorpus*, for domains of $2^n$ where $5 \le n \le 9$, using 80% and 20% of the corpus for training and validating, respectively.
2. Test the full classifier and the autoencoder using the full *TestCorpus*, for the five domain sizes;
3. For all memory registers of size $2^n\times 2^m$, where $5 \le n \le 9$ and $0 \le m \le 10$, fill up the whole of the *RemCorpus*, including all objects of the ten classes, and test the performance of the $\eta$-recognition operation using each object included in *TestCorpus* as the cue, by classifying all the retrieved objects and comparing the assigned class to that of the cue; then select the memory registers with the best number of arguments. Previous experiments show that values of $n \le 4$ are less satisfactory.
4. Test the performance of the $\eta$-recognition operation for the registers with $2^n$ columns selected in (3) and the best $2^m$ rows by filling them with different amounts of the *RemCorpus*, or entropy levels; select the register of size $2^n\times 2^m$ with the best performance.
5. Test the procedure with a standard tenfold cross-validation procedure.

The behavior of the system on this first experiment depends on the performance of the full classifier, on the memory size, and on the recognition’s parameters^3^. Here we use the setting in which the parameters’ values are $\iota = 0$, $\kappa =0$, $\xi =0$ and $\sigma =0.1$. The results of steps (1) and (2) of the procedure are presented in Tables 2 and 3. The former shows that the average accuracy of the classifier grows steadily up to the memory with 512 arguments, but the gain rate between memory sizes diminishes significantly; while the latter shows that the average root mean squared error (RMSE) of the autoencoder—scaled to a percentage of the maximum value of a pixel in the images (255)—shrinks steadily as the domain size doubles but, as in the case of the classifier, the gain diminishes greatly.

**Table 2:** Performance of classifier for different number of columns.

Number of arguments	Accuracy mean	Accuracy STD	Difference of means
32	70.2	8.6	0
64	80.8	0.6	10.7
128	85.9	0.6	5.0
256	88.2	0.5	2.3
512	89.3	0.3	1.1

**Table 3:** Performance of autoencoder for different number of columns.

Number of arguments	Scaled RMSE mean	Scaled RMSE STD	Difference of means
32	16.3	3.68	0
64	12.5	0.15	− 3.8
128	10.7	0.14	− 1.8
256	9.4	0.12	− 1.3
512	9.0	0.08	− 0.4

**Figure 6:**
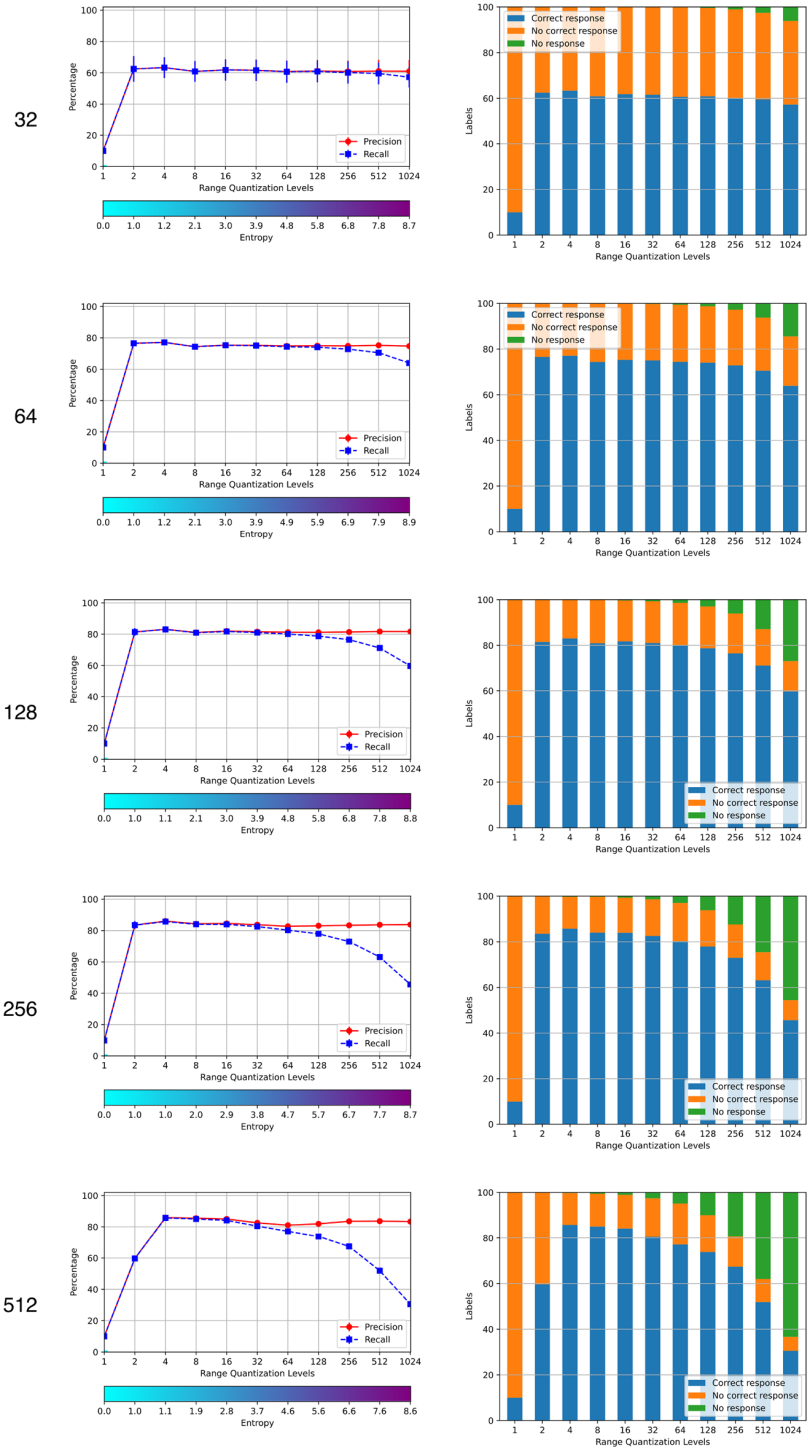
Precision and recall of the memory output for tables with with different sizes. The pair of graphs in each row correspond to the number of columns (arguments) shown on their left. The horizontal axis in each graph indicates the number of rows and the vertical axis indicates the percentage of precision and recall for the corresponding columns and rows.

The results of step (3) of the procedure are shown in Fig. 6, where the label *Range Quantization Levels* indicates the number of rows and the vertical axis indicates the percentage of the precision and recall for the corresponding columns and rows. All the objects in the *TestCorpus* belong to one of the ten Fashion-MNIST classes, so there are no true negatives. The output of the classifier may be a right response, a wrong response and no respond; the first corresponds to a true positive; the second to both a false positive of the selected class and a false negative of the right one; and the third means that the cue was rejected by the memory, so there was no retrieved object to be classified. Hence, in this setting, accuracy, precision and recall coincide if there is a response, while accuracy and recall decrease otherwise.

Figure 6 is consistent with Table 2, as the performance increases with the number of columns, but the gain decreases significantly too. The best performances for each number of columns are achieved with 2 to 32 rows, and correlate well with the ones obtained by the full classifier, as shown in Table 2. For example, the best accuracy of 85.7% for a domain of 256 arguments is achieved with a memory register of four rows, and corresponds to a full classifier accuracy of 88.2%. Hence, the memory contributes only to 2.5% of the total system error of 14.3%, achieving over 97% of accuracy at producing objects considered in the same class than the cue, with $\sigma = 0.1$.

**Figure 7:**
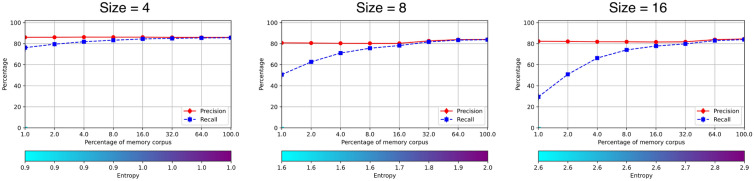
Performance of the memory recognition operation with different amounts of *RemCorpus*. The size value in each graph corrresponds to the number of rows of the register, as its number of columns has been set to 256.

We selected the memory with 256 columns for the rest of the experiments, as it provides a good compromise between performance and size. Next, we tested the performance of the memory for 4, 8 and 16 rows—which have the best performances—filling it with different amounts of the *RemCorpus*—1%, 2%, 4%, 8%, 16%, 32%, 64% and 100%, with the corresponding entropy levels, as shown in Fig. 7. In all three cases the memory is operative with only 16% of the remembering corpus, and the performance is sustained filling it with the whole of *RemCorpus*. Although the performance of the memory registers with the three number of rows is very similar at the operational range, the memory with 4 rows is slightly better, and the memory register of size $256 \times 4$ was chosen for the the experiments 2 and 3. This memory register is very small—i.e., $2^2\times 2^8=1024$ two bytes cells holding integer values—and yet can contain a huge number of remembered objects of the domain. The experiments 4 and 5 required a larger variability and the best results were obtained with 16 rows using 4 K bytes.

Figure 7 illustrates the entropy trade-off for memories with a satisfactory operational size. If the memory content is very low—and the entropy is very low as well—the cue must be very precise to match an object within the memory, but when it does, the selection is most likely right; conversely, if the memory is too dense and the entropy is too high most cues will match an object within the memory, but the selection will be often wrong; but whenever there is a moderate amount of memory content, cues are likely to match an object, which is often the right one. In the present illustration the operational range is quite large and AMRs with the three sizes have a satisfactory performance using only the 16% of the *RemCorpus*, and the precision does not decrease at the higher entropy level, suggesting that a much larger amount of objects can be registered in the memory.

### Experiment 2

**Figure 8:**
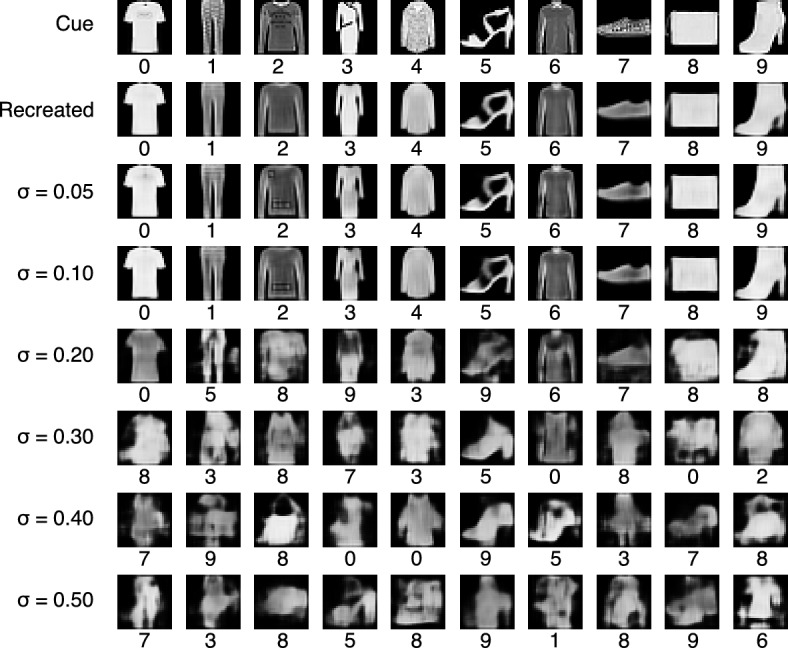
Recovered objects of all ten classes for different values of the parameter $\sigma$. The sample was elected randomly, on the condition to belong to the cases accurately processed by the classifier, and the class assigned by the classifier to the retrieved object is shown below the corresponding image. The first row shows the input cues; the second, the objects produced directly by the autoencoder; the remaining rows shows the images produced by the decoder from the memory outputs using distinct values for $\sigma$.

The register selected in Experiment 1 was filled up with the totality of the *RemCorpus* and the $\beta$-retrieval operation was performed using each object of the *TestCorpus* as the cue. Fig. 8 shows instances of cues—selected randomly, on the condition to belong to the 88.2% of cases accurately processed by the classifier—and the corresponding retrieved objects for the respective values of $\sigma$. The class assigned by the classifier to the retrieved object is shown below the corresponding image. The first row in Fig. 8 illustrates the input cues; the second, the objects produced by the autoencoder directly, which never rejects a cue. The third shows the reproduction of the cue with $\sigma = 0.05$; the fourth with $\sigma =0.1$, and so on until the last row, where $\sigma =0.5$. The objects reproduced by the autoencoder and recovered from the memory with a value of $\sigma$ up to 0.1 are visually similar and are assigned the correct class by the classifier, and can be thought as remembered objects. When $\sigma$ is 0.2, most of the constructed images look alike the input cue and are assigned the right class in four cases: T-shirt, Shirt, Sneaker and Bag (classes 0, 6, 7 and 8, respectively); the remaining six are assigned a different class: the trouser (class 1) retrieves a sandal (class 5); the pullover (class 2) a bag (class 8); the dress (class 3) an ankle boot (class 9); the coat (class 4) a dress (class 3); the sandal (class 5) an ankle boot (class 9); and the boot (class 9) a bag (class 8). These retrieval operations may be considered associations. For largest values of $\sigma$ the retrieved objects have a faint shape and are assigned a different class in most cases. Some resemble the cue vaguely; others resemble objects of a different class, others suggest compositions between objects, and some are too vague to be assigned an interpretation. In this latter condition the retrieved objects can be considered imaged objects or just noise.

### Experiment 3

**Figure 9:**
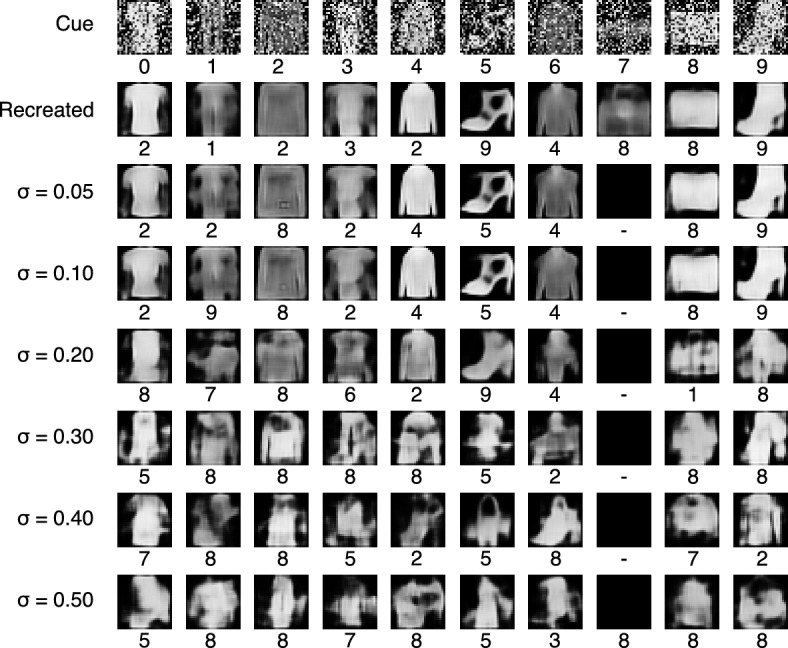
Recovered objects with noisy cues. The sample is the same as in Fig. 9, and the class assigned to the retrieved object by the classifier is shown below the corresponding image. The first row shows the input (noised) cues; the second, the objects produced directly by the autoencoder, while the remaining rows shows the images produced by the decoder from the memory outputs using distinct values for $\sigma$.

The third experiment is analogous to the second one but using incomplete cues. In our previous work we showed that the retrieved objects were satisfactory reconstruction of severely occluded cues^1,2^; in the present one we use cues with large amounts of noise. Half of the original pixels were randomly chosen, and their values were updated with random values—from 0 to 255. Figure 9 shows the results of this experiment. The cue and the output of the autoencoder are shown in the first and the second row, as before. In this case the autoencoder recovers the input image although with a lower quality and there are exceptions—e.g., the trouser (class 1) and the sneaker (class 7)—and half of the outputs are assigned the wrong class by the classifier—the t-shirt/top, the coat, the sandal, the shirt and the sneaker. The remaining rows show the objects recovered from the memory, as before. The black squares indicate that the cue was rejected and no object was retrieved, as was the case for the sneaker for all values of $\sigma$. Some of the objects recovered with $\sigma =0.05$ and $\sigma =0.1$ have the right shape and are assigned the correct class—the coat (class 4), the sandal (class 5), the bag (class 8) and the ankle boot (class 9)—and can be considered remembered, but the rest are assigned the wrong class and can be better thought of as associations or imaged objects. The objects recovered with values of $\sigma =0.3$ and $\sigma =0.4$ can be considered associated or imaged, and when $\sigma =0.5$ they may be imaged or just noise. Although the pictures have a lower quality than in the case with complete cues, the overall pattern of recovery increasing the value of $\sigma$ is sustained.

### Experiment 4

**Figure 10:**
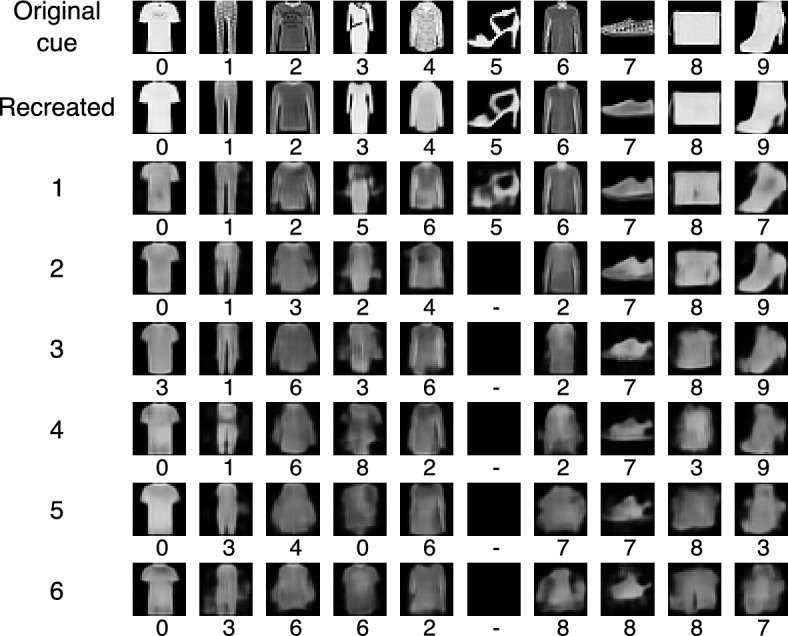
Associations chains from initial complete cues using an AMR of size $256 \times 16$ and $\sigma =0.15$. The sample is the same as in Fig. 8, and the class assigned by the classifier to the retrieved object is shown below the corresponding image. The first row shows the original cues; the second, the objects produced directly by the autoencoder; the third, the objects produced by the memory from the original cues, and the remaining rows show the ojects produced by the memory from the images in the previous row.

This experiment consisted on producing association chains starting up from a complete cue and using the retrieved object as a cue to a new retrieval operation, repeating the procedure recurrently. A form of this problem has been addressed within the ANNs literature but using a heteorassociative memory^44^. Figure 10 shows an association chain at each column, started with an object of each of the ten classes, using an AMR of size $256 \times 16$ and $\sigma =0.15$. The top row shows the initial cues; the second, the object produced directly by the autoencoder. The third shows the object produced by the $\beta$-retrieval operation using the top object as the cue; the fourth, the constructed object using the third as the cue; and so forth until a chain of length six is produced.

The classifier assigns the right class to the objects generated by the autoencoder and to seven objects retrieved from the memory using the original cue; the exceptions are the dress that is classified as a sandal, the coat as a shirt and the ankle boot as a sneaker. The visual shapes of all ten recovered objects are alike to the original cue, and the objects that have a different class assigned can be considered associations. The shape of the objects retrieved at the second time is also visually alike the cued objects although the chain of sandals is interrupted, as shown by the dark square. Six objects are also assigned the correct class but the pullover is classified as a dress, and the dress and the shirt as pullovers, which are also similar enough. The trend is sustained in the third retrieval operation, where four object are assigned a different class—the t-shirt is considered a dress, the pullover and the coat are classified as shirts, and the shirt as a pullover—which are visually similar and can also be seen as associations, although the quality of the images is lower and some are faint and vague. The pattern is continued further down the chain in the fourth, fifth and sixth recollection rounds, where the number of objects assigned a different class is increased by one or two at each turn, although the t-shirt/top recovers its initial class which sustains all the way to the last round; the sneaker is recognized as such in all but the last turn, the bag is assigned the original class in all but the fourth round, where is considered a dress, and the classifier assigns a class that looks similar to the original one in most cases, although some figures in the fifth and sixth recollection are faint and vague, and can be considered imaged objects or just noise.

### Experiment 5

**Figure 11:**
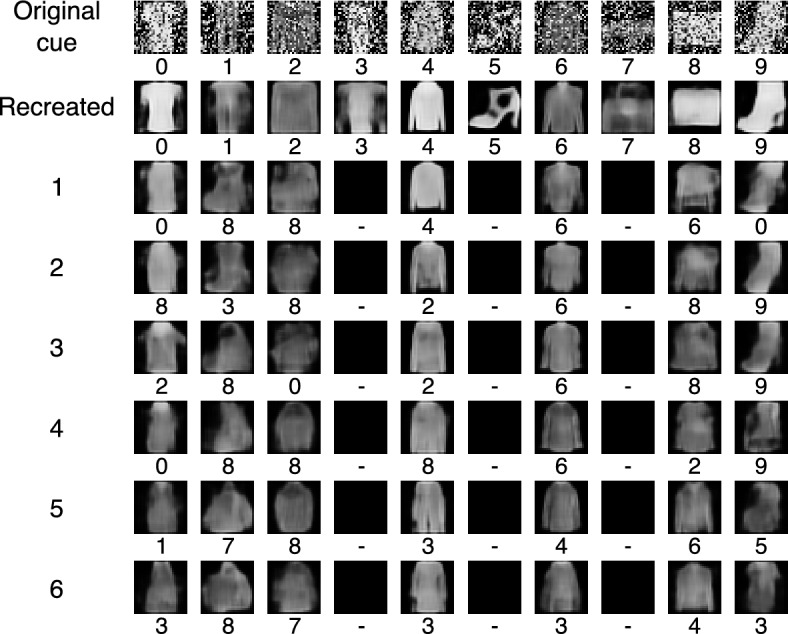
Association chains from initial noisy cues using an AMR of size $256 \times 16$ and $\sigma =0.15$. The sample is the same as in Figure 9, and the class assigned by the classifier to the retrieved object is shown below the corresponding image. As in Fig. 10, the first row shows the initial cues; the second, the objects produced directly by the autoencoder; the third, the objects produced by the memory from the initial cues, and the remaining rows show the objects produced by the memory from the images in the previous row.

This experiment is analogous to experiment 4—the AMR size is $256 \times 16$ and $\sigma =0.15$ too—but using noisy cues to start the association chains. Figure 11 shows that the autoencoder recovers the shapes with the right classes in all ten instances again, but memory retrieval is weaker than in the previous case. Most images are vague and faint since the first recollection; only the t-shirt/top, the coat and the shirt are assigned the right class, and the dress, the sandal and the sneaker are rejected directly, interrupting the corresponding remembering chains. The recollections of the remaining objects down the chain are vague and noisy, although some objects, such as the t-shirt, the coat and the shirt are visually alike the original cue, but are assigned related classes—the coat is classified twice as a pullover and twice as a dress; the shirt is assigned its right class up to the fourth cycle, and then is classified as a coat and a dress; the bag is classified as a shirt at the first turn, but back as a bag in the second and third rounds, although as a shirt, a pullover, a shirt and a coat in the remaining turns; and the ankle boot is also classified as such in the second, third and fourth round; but as t-shirt/top in the first, a sandal in the fifth and a dress in the sixth, although these latter images are rather noise. The overall pattern of the remembering chains is similar to the experiment with complete cues, although the images have a lower quality, and some chains are interrupted much earlier, as expected.

## Discussion

### Summary of experimental results

In experiment 1 we computed the optimal size of the memory register for the Fashion-MNIST, which is $256\times 4$ cells—each using 2 bytes holding integer values—as shown in Fig. 6. The remembered corpus consisted of 14,000 images—i.e., 20% of the 70,000 total images—all held distributed using only 2 K Bytes. The entropy is 0.9 when the memory is filled up with with only 1% of the *RemCorpus*, grows to 1.0 when 16% is included, and remains at that level all the way until the whole of remembered corpus is included, as shown in Fig. 7. As there are 14,000 explicitly registered objects and $e=1$, the productivity of the memory is $2^{256}-14,000$ emerging objects, providing a huge space for recognizing unseen inputs and retrieving novel constructions, but also false recollections.

Experiment 2 shows the impact of the parameter $\sigma$ on the retrieved objects of the ten classes of clothes, bags and shoes, as shown in Fig. 8. The images reproduced by the autoencoder and the ones recovered by the memory $\beta$-retrieval operation are similar to the input cue and are assigned the right class by the classifier up to a value of $\sigma$ of 0.1. For larger values of $\sigma$ the images are less similar and even noisy and often are assigned the wrong class. As $\sigma m$ is the standard deviation of the probability function $\zeta$ representing the cue, the smaller its value, the more reproductive or “photographic” the retrieved object in relation to the cue and vice versa. Experiment 3 shows the images produced when the cue is noisy. In previous work we showed that severely occluded objects can be reconstructed on the basis of incomplete cues and the memory content, and here we use noise instead of occlusions. The results of this latter experiment are analogous to the results of Experiment 2 although the images have a lower quality.

Experiments 4 and 5 show the production of association chains starting up with complete and noisy cues but using an AMR of size $256 \times 16$ and $\sigma =0.15$, as shown in Figs. 10 and 11, respectively. Preliminary experiments showed that the images produced using registers with only 4 rows were vague and faint from the start, and using 16 rows provided more variability to the argument’s values. The remembering chains where visually coherent although the quality of the imaged was diminished along the recollection cycles, as expected. The images generated with complete cues have a better quality than the ones produced out of incomplete cues, which was also expected. Objects recovered that have a good image quality and were assigned the class of the cue can be considered remembered. However, if they are assigned a different class can be considered associations; if the quality is diminished severely and the class varies, but can still be assigned an interpretation can be considered imaged objects; if these conditions are not met, the objects may be just noise.

The autoencoder never rejects a cue and its behavior opposes the $\eta$-recognition operation which rejects cues to objects not contained in the memory directly. The objects produced by the autoencoder in the second row should be copies of the cue—the decoder should compute the encoder’s inverse function—and both input and output should be of the same class. This condition is satisfied when the cues are complete, but there are exceptions when the cues are noisy—see Fig. 9—which is a known problem of objects synthesized by autoencoders. Memory retrieval may also produce similar objects of different classes, but as these are always constructions, the conceptual incoherence does not arise. However, it still remains the problem that visually similar synthesized objects can be assigned different classes. The present results suggest that such problem is due to the classifier’s precision, which is lower when classes are very similar, the images are vague, and the cues have poor quality or are noisy.

### Functional dissociation between storing and classification

In our previous work we used a designated associative memory register (AMR) for storing objects of the same class, and the memory system had a number of AMRs. In such architecture the $\lambda$-register operation was performed on the register corresponding to the class of the stored object and involved a form of supervised learning. We also showed that objects of different classes could be stored in the same register at the expense of a small increment of the entropy, and cues belonging to one class could retrieve objects of a different class. Nevertheless, these were very limited kinds of associations and storing and classifying were intertwined functions. In the present study we removed such restriction and used a single memory register for storing all the objects on a global medium. Hence, registering information is an unsupervised learning function; the stored objects are not classified within in the memory but classification is performed on the retrieved objects externally; and storing and classifying are conceptually and architecturally independent.

### Kinds of stored objects

The EAM system has been used to store manuscript digits and letters^1,2^ which are conventional visual signs, and its W-EAM extension to store and learn Mexican Spanish phones^3^, which are natural acoustic images. In the present experiment we used natural visual images of clothes and shoes, although with a very low resolution—the same used to store manuscript digits and letters. All these are instances of modality-specific images that have a spatial or a temporal extension, and are used as “units of remembering”.

### Indeterminacy and unconscious knowledge

The indeterminate character of the memory prevents inspecting the memory content independently of a cue. The indeterminacy also impacts on the number of objects stored—i.e., $2^{en}$—and seems to be a necessary representational property for holding the very large amount recollections of natural memories, which are hidden from conscious inspection within the storage medium, and yet can be made readily available when the memory is queried with a relevant cue. The relation between the indeterminate nature of memory and the determinate character of the objects retrieved through the $\beta$-retrieval operation can be illustrated with an analogy to the interpretation of ambiguous images, such as the famous duck-rabbit picture popularized by Wittgenstein in the Philosophical Investigations^45^. The ambiguous figure is a concrete image but the representation of the concepts of the duck and the rabbit are indeterminate. The memory retrieval operation recovers a concrete interpretation out of the concrete cue and the indeterminate representation, which may be the duck or the rabbit. However, the interpretation is unstable and easily faints and vanishes, but the process may be carried on recurrently. Another illustration is provided by the interpretation of emerging objects in geometric diagrams, as in the proof of the Theorem of Pythagoras^46^. The genuine indeterminate character of the human memory allows for storing a huge amount of unconscious knowledge, which can be probed and retrieved through appropriate cues. Such recollection may constitute real experiences, but one has to keep in mind that they may also be false recollections.

### The imagery debate

The present theory provides a computational notion of “image” and informs the imagery debate^47^. This debate confronts the view that all knowledge is propositional and has a linguistic character^48^ with the view that there are mental images^49,50^. The propositional side poses there is a language of thought using abstract symbols and an abstract syntactic structure, in which thought and reasoning are carried out^51^, while the imagery side holds that there is, in addition, a medium holding mental images which are inspected and manipulated directly by the mind’s eye and hand. In our model images do present the world to the computational agent but they are not the subject of mental manipulation. They are rather mapped into the corresponding amodal abstract representations at the second level as functions, which are mathematical objects, have a propositional character and may be used in thought processes, according to the intuitions of the propositional side, but functions are not symbolic expressions of an internal language. Such function may be abstracted away into the amodal indeterminate distributed representation stored in the declarative memory at the third level, and it is plausible that inference takes place by direct operations on such distributed representations, some of whose properties correspond to the intuitions underlying the imagery side, but these are not images; and it is also plausible that the mind uses both kinds of representations and their corresponding inference strategies. The heart of the debate seems to be that the propositional side does not allow for declarative–distributed–indeterminate–productive–constructive representations, while the imagery side acknowledges a form of them, although predicated on the images themselves rather than on the distributed representations from which they are produced.

### Natural declarative memory

Episodic^13^ and semantic memory^14^ are the paradigmatic forms of declarative human memory. These memories are relational, constructive and reject cues not included in the memory directly. In particular, episodic memory relies on a distributed memory network involving the hippocampus, the parahippocampal gyrus and the retrosplenial cortex, which supports not only memory register and retrieval but also is involved in the generation, maintenance and visualization of complex spatial context and imagination^15^. Cues are asserted and can be reinforced and strengthen, but episodic and semantic memory are not trained. Motor abilities are learned by repeated rehearsal but using procedural memory instead. The dissociation between registering episodic events and semantic facts, on the one hand, and acquiring abilities by rehearsing procedural memory, on the other, seems to be well established^13,14^. In our model this dissociation is reflected in the functional and architectural independence of the memory system proper, which is declarative, and the supporting coder, decoder and classifier, which are procedural. An autoencoder can be seen as a procedural memory because it reproduces the input cue, and action schemes, motor and linguistic, can be modeled with such kind of devices. However, the declarative memory augments the power of the cognitive agent as the recollections can be used on demand by language and thought process, independently of the schematic machinery provided by procedural memories. Episodic and semantic memory involve a larger functionality that the declarative machinery alone; for instance, episodic memory relates recollections with the self and involves the subjective sense of time to codify the autobiography of the agent^13^; and the semantic memory stores meanings rather than episodes, and has independent components, such as the terminological and the encyclopedic knowledge, that need further levels of structure. It is also plausible that there are several declarative memory modules associated to specific kinds of knowledge, such as a phonological memory, as suggested in our previous work^3^, the mental lexicon, and declarative memories for faces, shapes, and other kinds of objects of common experience.

### Perspectives and future work

One goal of our research program is to store more complex images that have spatial and temporal extension, and a multimodal character, such as videos and movies, which can in principle be represented as a single function with designated arguments for each of the modalities involved, coded and decoded with the corresponding modality-specific coder and decoder modules. Such representations could include features of other modalities of perception, as the somatic, gustatory and olfactory, and even internal arguments of the computational agent, such as the emotional state associated to registering the images. The functions at the second abstract amodal level of representation can be seen as a features-value structures including segments for each modality, and total or partial cues to the $\eta$-recognition and $\beta$-retrieval operations can be used to recognize and recover multimodal images that constitute units of remembering. Once such complex images are stored can be retrieved using different values of $\sigma$ and cues would be reconstructed as remembered, associated or imaged objects, as shown for clothes, bags and shoes in the present study. Likewise, chains of recollections can be established by using the recovered object as the cue to a new retrieval operation recurrently. Such images would lower their quality along the recollection chain and would be perceived as faint but novel reconstructions or imaged objects, possibly with a spatial and temporal extension. We also leave for future research the composition of images and the production of more general association chains, and the extension of the model to the hetero-associative case.

## Experimental setting

The experiments were programmed in Python 3.10 on the Anaconda distribution. The neural networks were implemented with TensorFlow 2.10.0, and the graphs were produced using Matplotlib, ImageMagik, and Inkscape. The experiments were run on an Alienware Aurora R5 with an Intel Core i7-6700 Processor, 16 GBytes of RAM and an NVIDIA GeForce GTX 1080 graphics card.

## Data Availability

The dataset used in the present study is available at https://github.com/zalandoresearch/fashion-mnist. The full code and the detailed experimental results are available at https://github.com/eam-experiments/fashion.
